# Intelligence Types Predict Different Domains of Emotional Creativity Through Self-Assessed Creativity and Intelligence

**DOI:** 10.11621/pir.2025.0104

**Published:** 2025-03-01

**Authors:** Tatiana V. Kornilova, Maria A. Chumakova, Lydia B. Maksarova

**Affiliations:** a Lomonosov Moscow State University, Russia; b National Research University ‘Higher School of Economics’, Moscow, Russia

**Keywords:** intelligence, emotional intelligence, emotional creativity, implicit theories, self-assessed intelligence, self-assessed creativity, TEIQue-SF, ECI

## Abstract

**Background:**

This study is based on the idea of unity between affect and intelligence. It explores how different types of intelligence (fluid, verbal, self-assessed, and emotional) contribute to emotional creativity and implicit theories of creativity and emotion.

**Objective:**

To identify the contributions of various types of intelligence to emotional creativity and the implicit theories of creativity and emotion.

**Design:**

Linear regression and mediation analyses were conducted on a sample of 244 students. These analyses aimed to uncover the relationships between different types of intelligence, emotional creativity, and related components of self-awareness (at the levels of self-assessment and implicit theories).

**Results:**

The findings demonstrated the intricate connections between various forms of intelligence-fluid, crystallized, emotional, emotional creativity, and self-awareness components such as self-assessment of intelligence and creativity. Specifically, the impact of intelligence on emotional intelligence traits and emotional creativity was mediated by measures of self-esteem in intelligence and creativity. To test specific hypotheses, we conducted a series of regression analyses and developed two structural models. The first model included linear regression equations with each emotional creativity (EC) scale as the dependent variable and both types of intelligence, as well as self-assessments — SAI and SAC — as predictors. The second model demonstrated the mediation effect of implicit theories of emotions (ITE) in the influence of emotional intelligence (EI) on emotional creativity.

**Conclusion:**

This study highlights the complexity of the interplay between different types of intelligence and emotional creativity. It reveals the mediating role of self-esteem in these relationships and underscores the distinct nature of emotional creativity domains. Additionally, it establishes the link between implicit theories of emotions and creativity, with emotional intelligence and self-esteem, offering valuable insights for further research.

## Introduction

Recent studies of emotional intelligence, emotional creativity, and implicit theories (of intelligence, creativity, and emotions) highlight the idea of unity between intelligence and emotion (B. Spinoza, L. S. Vygotsky), not only in their cognitive and personality components, but also on the different levels of mental processes. Self-assessments reflect the self-awareness level of an individual, where cognitive processes interact with those pertaining to emotions and personality, while the deeper levels of mental organization are represented by implicit theories. Implicit theories are a person’s subjective ideas about their intellect, personality, creativity, family, etc., which are less conscious, since they are formed and developed spontaneously in individual experience and manifest themselves in personal beliefs (mindset) and behavioral regulation (Dweck, 2006; Petrides et al., 2016; Sternberg et al., 2000).

Reviewing the latest meta-analysis of the relationship between intelligence and creativity (Gerwig et al., 2021), a meta-analysis of the relationship between emotional intelligence (EI) and creativity (Xu et al., 2019), and other works in this area, has revealed that the role of both types of intelligence — academic (IQ) and emotional (EQ) — in emotional creativity (EC) is not discussed. Emotional creativity is leftout when considering possible moderators of creative achievements. In our opinion, the confirmed connections between intelligence and creative achievements cannot be automatically transferred to those between intelligence and creativity in generation of emotions. Therefore, it becomes relevant to identify the role of both types of intelligence in emotional creativity (EC), as well as their common relationships with individual characteristics at the metacognitive level of an individual’s self-awareness (specifically, with self-assessment of intelligence and creativity).

What has been overlooked in the debate about general or domain-specific creativity, the four or seven C’s, is the system of interconnected multi-level processes that contribute to emotional creativity. The authors of a recent meta-analysis on emotional creativity note that “EC has been found to be related to various constructs across different fields of psychology during the past 30 years, but a comprehensive examination of previous research is still lacking” (Kuška et al., 2020, abstract).

Since there is no consensus on whether EC should be understood as an ability or a trait, and given that EC is assessed using a questionnaire, we considered it appropriate to use a questionnaire to assess emotional intelligence as well. This choice led us to approach the components of EI as personality traits, though distinct from the traits covered in the Big Five model (Petrides, 2009).

The purpose of this paper was to perform a comprehensive analysis of links among academic intelligence — both crystallized and fluid, self-assessed intelligence, self-assessed creativity, emotional intelligence, emotional creativity, implicit theories of creativity, and emotions. Given the multiplicity of possible connections, our focus was primarily on elucidating how traits of emotional intelligence, self-assessed intelligence, self-assessed creativity, and implicit theories of creativity and emotions contribute to emotional creativity.

### Emotional Intelligence — Ability or Trait?

While some authors question the theoretical basis of the emotional intelligence construct (Hughes & Evans, 2016), this construct has become integrative for building bridges between intelligence and personality research. Both public and academic interest in emotional intelligence (EI) have been rising since the 1990s, leading to the development of two main approaches to conceptualizing, defining, and measuring EI: as an ability — within the four-branch model by J. Mayer, P. Salovey, and D. Caruso (Mayer et al., 2016) — or as a personality trait.

There are studies that correlate EI ability with crystallized and fluid intelligence in different ways. MacCann (2010) found stronger associations of EI with crystallized intelligence (*r* = .71) than with fluid intelligence (*r* = .45). Some authors (Davies et al., 1998) understood EI as part of the fluid intelligence associated with the operational capabilities of the human cognitive system, while others (Farrelly & Austin, 2007), on the contrary, understood EI as part of crystallized intelligence, reflecting the ability to use knowledge. However, the negative relationship between EI and intelligence indicated that EI could not represent a new kind of intelligence (Husin Wan et al., 2013).

R. Bar-On proposed a model of emotional and social intelligence as emotional competence and introduced the ‘emotional quotient’ (EQ) index by analogy with cognitive IQ (Bar-On, 2007). Over the past decade, the understanding of EI as a personality trait began to be considered in the context of research by A. Furnham and K. Petrides. They substantiated the understanding of EI as a trait and insisted that this additional dimension does not fit either the model of H. Eysenck’s Big Three traits (extraversion, neuroticism, psychoticism) or the Big Five model (Petrides et al., 2016; Petrides & Furnham, 2003). Unlike other personality traits, EI traits are relegated to lower levels of awareness, but are reflected in emotion-related self-perceptions and personality predispositions regarding emotional regulation (Petrides, 2009; Petrides et al., 2018; Petrides & Furnham, 2003).

D. Van der Linden and co-authors showed in their meta-analysis that EI as a trait demonstrated significantly stronger associations with the General Factor of Personality (GFP) than with measures of EI as an intellectual ability, and this finding became one of the reasons for considering EI mostly as a trait (van der Linden et al., 2017).

Nevertheless, EI measured as ability, but not EI as a trait, is positively associated with performance of ‘hot’ cognitive tasks (Gutiérrez-Cobo et al., 2016); it seems to particularly contribute to cognitive processes when emotions are involved in the problem solving (Checa & Fernández-Berrocal, 2019). Such evidence allowed J.M. Mestre and colleagues to consider including EI ability in the Cattell-Horn-Carroll model, which is currently the most comprehensive and psychometrically validated model of the structure of cognitive abilities (Mestre et al., 2016). However, the proposal to include EI trait as a moderator of the relationship between EI ability and emotion regulation to provide greater subtlety in understanding emotion-related behavior (Hughes & Evans, 2016) was not supported. Mestre highlights the problematic term “EI trait”: both theory and empirical evidence show that EI trait assessment tools measure personality rather than intelligence.

The process in which abilities and personality traits converge in the context of EI is emotional regulation. The study by M. Bucich & C. MacCann (2019) found no relationship between of EI as an ability and everyday emotion regulation. However, as a trait, emotional intelligence is associated with various emotion regulation strategies such as social sharing, direct situation modification, and reappraisal. The results of the meta-analysis showed that emotional regulation is more strongly associated with trait-based measures of EI than with ability-based measures (Peña-Sarrionandia et al., 2015).

### Emotional Creativity

The term “creativity” refers to both cognitive abilities and personality traits, creating the same dichotomy as in EI. Previous research that examined relationships among EI, intelligence (divergent thinking), and creativity indicated that cognitive abilities positively, but not significantly, correlated with creative abilities and creative achievements (Kim , 2008; Said-Metwaly et al., 2022; Weiss et al., 2021); however, both academic intelligence and creativity significantly negatively correlated with EI trait estimates (Furnham, 2016).

Currently, the assessment of EC traditionally relies on the questionnaire developed by J. Averill, who introduced the concept using a constructivist approach (Averill, 1999, 2000). He considered the interaction of cognitive and emotional processes as integral emotional syndromes that vary depending on the culture (Averill, 2005). A social constructionist view reveals a new aspect of emotion regulation — the process of construction as a moment of creativity and invention of a new, effective, and authentic emotional reaction. In this approach, we can observe similarities with the development of the L.S. Vygotsky’s idea about the mediating function of self-regulation (Vygotsky, 1980). We believe that the stimuli-tools necessary for emotional self-regulation are not the words themselves (or the rules set by society), but the active manifestation of a person’s creative potential. The term “emotional creativity” refers to a novel form of creativity, where the outcome is a new emotional reaction.

In Averill’s model, EC included three components: cognitive skills, emotions, and creativity. Emotional schemes assimilated in society belong to the cognitive component in the EC. For empirical studies of EC, the Emotional Creativity Inventory (ECI) was developed (Averill, 1999). The revealed connections of EC with academic and emotional intelligence support its interpretation as an emotional ability (Averill, 2000). Research has shown that women demonstrated significantly higher levels of EC on all scales than men (Kuška et al., 2020).

EC correlates with different types of creative activity: writing, drawing, performing a dramatic role, composing music, and home improvement (Trnka et al., 2016). In addition, the works of participants with higher EC scores are rated as more creative. Emotional creativity is also associated with the Big Five “openness to experience”, including the “aesthetic” facet (Averill, 1999).

In our previous study, the modified Russian version of the ECI revealed five latent factors (Kornilova et al., 2020). The first two factors — Novelty and Emotional Preparedness — were similar to those identified by Averill. The Effectiveness and Authenticity of emotional reactions formed two factors (instead of one in the original version), indicating greater differentiation in Russian-speaking samples. Finally, the fifth factor was unique for the Russian sample and included items not only about the variety of emotions experienced, but also about the complexity of their expression (Emotional Diversity).

### Implicit Theories of Emotions

Implicit Theories (IT), or Lay Theories, are understood as preconscious or partially conscious ideas about various phenomena, which, based on developing internal schemes (for example, tacit knowledge), set the possible ranges of the subject’s reaction to a specific event and attitudes to specific situations. Implicit personality theory is not a theory in the scientific sense, but rather a set of ideas about one’s own personality and the personalities of others. Similarly, the Implicit theory of intelligence refers to everyday ideas about intelligence, as contrasted by R. Sternberg with the explicit theories of professionals (Sternberg et al., 2000).

In the context of clarifying the connection between emotional intelligence and emotional creativity, we are primarily interested in implicit theories of emotion (ITE), which reflect a person’s everyday ideas about whether emotions help or hinder in achieving goals and whether a person can control their emotions (Karnaze & Levine, 2020).

The division of implicit theories into incremental and constant (Dweck, 2006) has been extended to the field of study of implicit theories of emotion. M. Tamir and her colleagues developed the ITE diagnostic scale (Tamir et al., 2007). Studies have shown that adhering to incremental ITE (as opposed to constant ITE) is linked to greater effectiveness in emotional self-regulation. This includes the use of productive strategies, for example, cognitive reformulation and cognitive reappraisal, and the rejection of unproductive strategies, like the suppression of emotions (De France & Hollenstein, 2021). Incremental ITE is also associated with greater motivation to make efforts to self-regulate emotions, with less negative emotions and pathological distress (Kneeland et al., 2016), as well as with greater levels of positive emotions, social support, and subjective well-being (De France & Hollenstein, 2021; Ford & Gross, 2019; Kneeland et al., 2016; Romero et al., 2014).

However, despite active research into IT, we have not found any studies on the association of ITE with EC scores. Based on previous research, it can be assumed that adherence to incremental ITE is linked to higher levels of emotional intelligence and emotional creativity.

### Self-Assessed Intelligence (SAI) and Self-Assessed Creativity (SAC)

Studies examining self-assessed intelligence and self-assessments of cognitive ability across various samples have shown significant, albeit moderately sized, correlations between self-evaluations and performance-based indicators of constructs associated with intelligence (Howard & Cogswell, 2018). Although intellectual ability is one of the major predictors of SAI, the mapping between the two is far from perfect. Part of this measurement error or inaccuracy is likely due to the effects of IT. Differences between IT types corresponding with SAI inaccuracy are captured by a more integrative concept of the mindset: whether or not the person tends to view their and others’ intelligence as a relatively fixed or a malleable trait (Dweck & Yeager, 2019).

Creative self-esteem correlates with a positive prediction of creative self-regulation, which is a major factor in creative activity, achievement, and future participation in creative endeavors (Zielinska et al., 2022). Self-reporting tools can provide insightful detailed information about a person’s perception of creativity, including their own (*e.g.*, the J. Kaufman Domains of Creativity Scale K-DOCS (McKay et al., 2017)). We s hare Kaufman’s position that different components are included in the self-assessment of creativity: actions, evaluation, process, and beliefs (Kaufman, 2019). General self-assessment of creativity reflects how a person perceives their creative thoughts and processes (Silvia et al., 2012).

Creative self-esteem is based on a creative self-concept, but to a greater extent includes emotional components and a value attitude towards oneself. Thus, creative self-esteem should be viewed as a mediating variable between the domains of cognition and personality (“affect” and “intelligence”).

The connections between SAI and SAC with a person’s ideas about the ability to control emotions (preferences to incremental or constant ITE) are the least studied.

### The Research Problem Statement

The present study was motivated by the lack of data on the contribution of creative self-esteem, ITE, and intelligence to emotional creativity (EC). While previous research has explored the links between IQ and EI, as well as between intelligence, EI and EC, links between intelligence and SAI, links between EI and emotional regulation (Geher et al., 2017; Gutiérrez-Cobo et al., 2016; Furnham, 2016; Howard & Cogswell, 2018; Hughes & Evans, 2016; Kornilova et al., 2009; et al.), most studies have mainly examined pairwise combinations of these constructs or included the third construct as a moderator. But it is difficult to find a study that integrates the relationship between the measurements of these constructs on the same sample.

Also, the basis for our study lies in the inconsistency of associations between intelligence and emotional intelligence reported in the literature. Variations in data are observed depending on the country and the methods employed (Husin et al., 2013; Sanchez-Ruiz et al., 2011). Therefore, it is necessary to reassess these relationships each time when discussing the impacts of these variables on emotional creativity.

Another important motivation for us was to distinguish between studies of EC in the context of the paradigm proposed by Averill and studies of the influence of mood and emotions on creativity in the context of creative performance (*e.g.*, Baas, 2019). The susceptibility of creativity to contextual affective factors has already been studied. For emotional creativity, the key research question is formulated not about the influence of emotions on it, but about the contribution to the generation of emotions from multiple processes of both cognitive and personal regulation. In this case, the level of self-assessments can be understood as a higher level of regulation in comparison with EI, and the level of implicit theories as a lower one. An important aspect of regulation at the level of self-assessments is a more pronounced saturation of SAC with personality components, as suggested by other authors (Beghetto & Karwowski, 2017; Kaufman, 2019; et al.). Accordingly, we can expect the closest relationship between SAC and EI as a personality trait, and not as an ability.

We are not aware of works where intelligence, EI, EC, and ITE, and SAI and SAC are considered in a comprehensive manner on the same sample, which would help clarify the regulatory role of both cognitive and personality processes combined. Our general assumptions were that the variables of intelligence and emotional intelligence contribute to emotional creativity; that both the level of self-concepts and the level of implicit theories involve processes that mediate emotional creativity. Generalization of the available data on the relationships of the studied constructs allowed us to formulate the following hypotheses:

*Hypothesis 1.* Intelligence is positively associated with emotional intelligence (EI), with self-assessed intelligence (SAI) and creativity (SAC), and both types of intelligence predict emotional creativity (EC) and self-assessed intelligence and creativity;

*Hypothesis 2.* SAI and SAC are connected with each other and directly predict EC;

*Hypothesis 3.* SAI and SAC mediate intelligence impact on EC;

*Hypothesis 4.* Implicit Theories of Emotions (ITE) mediate the impact of EI on EC;

*Hypothesis 5.* An integrative model of the impact of different types of intelligence on emotional creativity, including the mediator effects of implicit theories of emotions and self-assessments of intelligence and creativity, more accurately reflects the structure of relationships between the studied constructs compared to a complex regression model.

## Methods

### Participants

A total of 244 students (203 females) majoring in psychology at Lomonosov Moscow State University aged from 17 to 47 were recruited for the study. Our sample turned out to be quite young (*M* = 20.01; *SD* = 2.71) and predominantly female. At the same time, we found a medium-sized effect of age differences between males and females (M_f_ = 19.76, SD_f_ = 2.31; M_m_ = 21.22, SD_m_ = 4.00; Hedges’ *g* = .55).

All the participants were administered a set of questionnaires and self-assessment inventories; 155 respondents also completed an intelligence assessment.

The model was built based on the data from 127 women and 25 men (excluding participants with missing data).

### Procedure

*Fluid Intelligence (FIQ)* was assessed using two subtests from the freely distributed test battery ICAR (International Cognitive Ability Resource; Condon & Revelle, 2014) in the Russian-language approbation (Kornilova et al., 2019). The subtests contain 24 3D shapes that require mental rotation and 11 matrices that require problem solving. The 3D figures per rotation are cubes, and participants are asked to determine which of the six suggested answers is a possible rotation of the cube presented.

The task with matrices is similar to Raven’s progressive matrices. Stimuli are geometric figures of 3 x 3 elements with one of the nine elements missing. Respondents must identify which of the six suggested elements would best complete the figure. FIQ test score was calculated as a weighted sum of correct answers for both subtests.

*The Assessment of Verbal Intelligence (VIQ)* was conducted using two subtests developed as a part of the ROADS battery (Kornilov & Grigorenko, 2010). The first subtest is an analogue of the Mill-Hill test and includes 34 tasks to determine, among the six proposed words, the closest in meaning to the given word. The second subtest — Analogies — includes 30 tasks to establish synonyms / antonyms between pairs of words. The VIQ score was calculated by summing the equally weighted scores for these subtests.

*Trait EI* was assessed by the TEIQue-SF (Petrides, 2009) in Russian adaptation (Kornilova, 2023). The Cronbach’s α coefficients for TEIQue-SF scales: α = .87 for the Well-Being scale, α = .79 for the Decreased Self-Control scale, α = .66 for the Emotionality scale, and α = .75 for the Sociality scale.

*EC* was assessed by Averill’s Emotional Creativity Inventory (ECI) in Russian adaptation (Kornilova et al., 2020). The Cronbach’s α for all scales exceeded .70.

We included subtest scores on the TEIQue-SF and ECI questionnaires (rather than a single score on EI or EC) in our data analysis, as they reflect specifically focused domains in the constructs measured by the questionnaires.

To assess *ITE*, we used the M. Tamir scale with four questions, two of which refer to the constant implicit theory of emotions, about the impossibility of controlling them, and the other two refer to the incremental ITE, suggesting the possibility of voluntary control of emotions (Tamir et al., 2007). The one-factor solution of the constructed questionnaire with a positive pole for the incremental theory and the Cronbach coefficient α = .69.

*SAI* and *SAC* were assessed through the direct self-assessment procedure described by A. Furnham (Chamorro-Premuzic & Furnham, 2006). It involved presenting a bell curve with a range of scores from 55 to 145 and instructing the participant to score themselves.

*Data processing*. The study tested the hypotheses using a combination of correlational and multivariate regression analyses, and structural equation modelling (SEM) using R programming language, an open-source language used for statistical computing or graphics (R Core Team, 2022), implemented in RStudio Version 2023.03.0+386.

## Results

### Descriptive Statistics

Descriptive statistics showed that in our sample, both intelligence scores were slightly higher in men who were older. Indices on the emotional intelligence scales of Well-Being and Sociality were slightly higher in women, and the Emotionality index was slightly higher in men. For the Novelty EC scale, the scores for women were slightly higher, and for the Emotional Diversity scale, they were moderately higher. No differences were obtained for ITE, SAI, and SAC.

**Table 1:** Descriptive Statistics for Assessed Scales with Effect Size for Gender Differences

	Male		Female			
Scale	*M*	*SD*	*M*	*SD*	*Hedges’ g*	*Effect*
*Intelligence*						
FIQ	10.20	14.07	96.53	12.98	.28	Little higher in men
VIQ	97.12	14.02	91.69	15.02	.37	Little higher in men
*Emotional Intelligence*						
Well-being	4.00	8.35	42.44	8.80	.28	Little higher in women
Emotionality	17.68	4.42	16.53	5.55	.21	Little higher in men
Decreased Self-Control	2.29	5.21	2.72	6.17	.07	No effect
Sociality	44.07	7.21	45.62	7.27	.21	Little higher in women
*Emotional Creativity*						
Authenticity	13.00	1.81	12.93	2.51	.03	No effect
Effectiveness	21.30	4.52	21.43	4.50	.03	No effect
Emotional Preparedness	22.48	3.19	22.50	3.07	.01	No effect
Novelty	17.90	5.69	19.57	5.34	.31	Little higher in women
Emotional Diversity	18.98	4.56	21.82	4.99	.58	Moderately higher in women
*Implicit Theories of Emotions*						
ITE	3.84	.65	3.74	.77	.13	No effect
*Self-Assessments*						
SAI	111.72	11.01	11.77	14.65	.07	No effect
SAC	109.90	18.82	106.65	2.07	.16	No effect

We performed a series of linear regression analyzes with Benjamini-Hochberg correction for Type I error in the significance of the F-statistics. In every regression model one variable was the dependent variable, and gender, age, and their interaction were included as predictors. The results presented in *Appendix Table A* show that the regression models for FIQ, VIQ, EI, ITE, SAI, and SAC did not fit the data: all *p*-values for F-statistics were significantly higher than .05 and all adjusted *R*^2^ were close to or less than .

Four of five EC scales were independent of gender and age impacts. However, we obtained those impacts for Emotional Diversity (F(df) = 7.80(3/239), *p*(F) < .001, adjusted *R*^2^ = .08): females demonstrated higher scores and age was a significant negative predictor of scale scores.

To avoid mixing the described effects of age and gender with the interaction between the studied variables, in further analysis of Emotional Diversity we used regression residuals from the constructed regression model instead of scale scores^1^. Other variables were recalculated using z-transformation to ensure the uniformity of measurements.

### Correlation Analysis

We performed a correlation analysis using Pearson’s correlation coefficient with Benjamini-Hochberg correction for Type I error. The matrix is presented in *Appendix Table B.*

#### Intelligence and Emotional Intelligence

VIQ was positively associated with FIQ (r = .26, p = .006), FIQ with SAI (r = .25, p = .007), SAI was positively associated with SAC (r = .43, p < .001), but SAC was not significantly associated with intelligence domains. We can accept Hypothesis 1 regarding the relationship between intelligence and self-assessments, but only for SAI, and not for SAC. The relationship between intelligence and emotional intelligence, assumed in Hypothesis 1, was not confirmed.

Intelligence was not associated with EC in most of its dimensions: only a negative correlation of VIQ with the Novelty scale was found (*r =* –.22, *p =* .019). Whereas all scales of EI were significantly correlated to the EC scale of Effectiveness. We obtained positive correlations for Well-Being (*r =* .41, *p <* .001) and Sociality (*r =* .39, *p <* .001) and negative correlations for Emotionality (*r =* –.29, *p <* .001) and Decreased Self-Control (*i.e.*, positive with increased self-control) (*r =* -.27, *p <* .001). The EC scale of Novelty was positively correlated with Sociality (*r =* .17, *p <* .001).

#### Implicit Theories of Emotions

Adherence to the incremental ITE correlated with two scales of EI: positively with Sociality (r = .21, *p* = .006) and negatively with Decreased Self-Control (r = –.27, *p* < .001). Also, ITE was related to the EC scale of Effectiveness (r = .19, *p* = .010).

#### Self-Assessments Relations with EI, ITE, and EC

SAC positively correlated with EI scales of Sociality (r = .22, *p* = .002) and EC — Novelty (r = .19, *p* = .010). SAI was also associated with the EI scales of Well-Being (r = .16, *p* = .046) and Sociality (r = .23, *p* = .001) and negatively with the Decreased self-control (r = –.16, p = .046). Also, SAI was positively associated with two of the five EC scales: Emotional Preparedness (r = .18, *p* = .019) and Emotional Diversity (r = .16, *p* = .033).

#### Intelligence and Emotional Intelligence Predict Emotional Creativity and Self-Assessment

To test more complex hypotheses, we reduced measurement dimensions for the EI data using exploratory factor analysis. KMO test for EI scales showed high suitability for factor analysis (overall MSA = .79, MSA for scales varied from .77 to .81). We performed maximum likelihood factor analysis to extract one factor that unified the TEIQue scales (TLI = 1.024, 47 of variance explained) and calculated factor scores for each participant as an integral measure of the EI trait.

We performed a series of linear regression analyses with Benjamini-Hochberg correction for Type I error in the significance of the F-statistics. In every regression model, EC scales, SAI, and SAC were the dependent variables, and FIQ, VIQ, and the integral measure of the EI trait were included as predictors. The results presented in *Appendix Table C* showed that the regression models for Authenticity, Emotional Preparedness, Novelty, and Emotional Diversity did not fit the data.

We obtained a significant positive effect of Emotional Intelligence on the EC Effectiveness scale (β = .51, t = 5.96, *p(t*) < .001), a significant positive effect of fluid intelligence on SAI (β = .22, *t =* 2.80, *p(t*) = .006), and a significant positive impact on SAC of both verbal (β = .17, *t =* 2.08, *p(t*) = .040) and emotional (β = .22, *t =* 2.50, *p(t*) = .014) intelligence. We can accept Hypothesis 1 with limitations concerning the relationship between Emotional Intelligence and Emotional Creativity, as well as between types of IQ and Self-Assessed Intelligence.

#### Self-Assessments Predict Emotional Creativity

**Figure 1:**
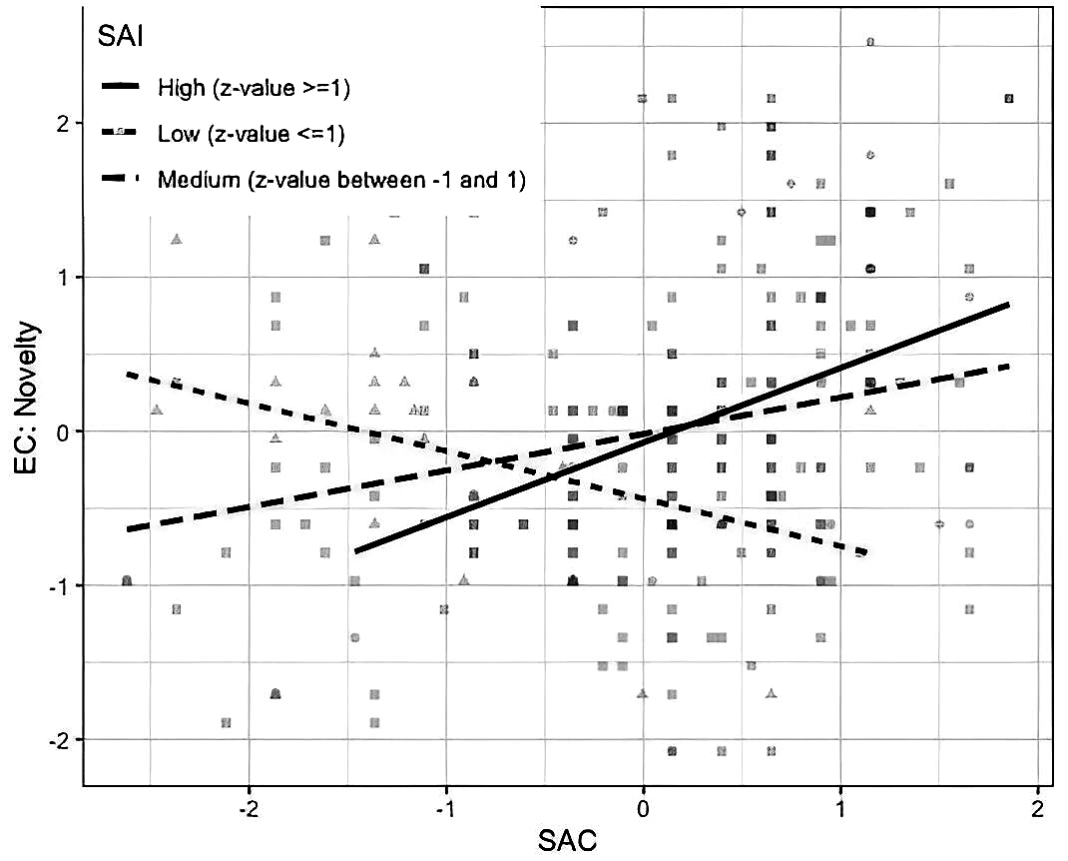
SAI and SAC effects on EC Novelty scale

We performed a series of linear regression analyses with Benjamini-Hochberg correction for Type I error in the significance of the F-statistics. In each regression model, each of the EC scales was the dependent variable, and SAI, SAC, and their interaction were included as predictors. The results presented in *Appendix Table D* showed that a significant effect of SAI and SAC was found only for the EC Novelty scale. *Figure 1* demonstrates the direction of obtained effects. For participants with medium and high self-assessed intelligence, Novelty scores increased in proportion to SAC scores, whereas for participants with low SAI, we obtained the opposite effect.

#### SAI and SAC Mediate Intelligence Impact on Emotional Creativity

**Figure 2:**
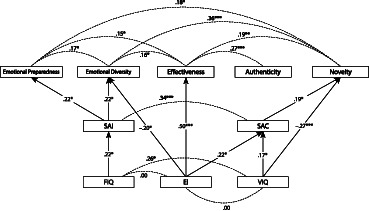
SAI and SAC mediate Intelligence impact on Emotional Creativity

Path analysis was performed using structural equation modeling implemented in the lavaan package in R (Rosseel, 2012). We used maximum likelihood estimation with the NLMINB optimization method. Missing values in the data were listwise deleted. The model tested included linear regression equations with each of the EC scales as the dependent variable and both types of intelligence, SAI and SAC, as predictors. Additionally, we specified in the tested model expected covariations between different types of intelligence and between self-esteem. The tested model demonstrated satisfactory fit indices: χ2(df) = .924 (2), p = .63. In *Figure 2* we report only significant estimations for obtained effects.

Our results showed that in a complex model that included both the levels of various types of intelligence and the levels of self-esteem, new specific relationships between the studied variables were revealed. First, we verified the effect of FIQ on SAI, the effect of VIQ and EI on SAC, and the effect of EI on the EC Effectiveness scale. Second, we verified the effect of SAC on the EC Novelty scale. The new findings compared to the previous analysis results were positive SAI effects to EC scales of Emotional Preparedness and Emotional Diversity. In addition, our analysis showed that verbal intelligence had a negative direct effect on the EC Novelty scale, but a positive indirect effect on scores on this scale through SAC, which was also positively affected by EI. Thus, we confirmed Hypothesis 3 about the mediation effect of self-assessments of intelligence and creativity on the contribution of Intelligence to Emotional Creativity. We can argue that the Emotional Intelligence trait and Verbal Intelligence had both direct and indirect effects on Emotional Creativity, while Fluid Intelligence had only an indirect effect on Emotional Creativity through the level of Self-Esteem.

#### Implicit Theories of Emotions Mediate Emotional Intelligence Impact on Emotional Creativity

We performed path analysis using the same technique as in the previous analysis. Additionally, we specified in the tested model expected covariations between different types of intelligence.

**Figure 3:**
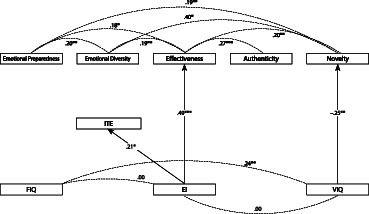
Implicit Theories of Emotions (ITE) mediate EI impact on Emotional Creativity

The tested model demonstrated satisfactory fit indices: c^2^(df) = 1. 124 (2), *p =* .57. In *Figure 3,* we report only significant estimations for obtained effects. Our analysis revealed no significant mediation effects of ITE. However, we obtained expected positive EI effect for the Effectiveness scale and positive EI effect for the ITE scores. Thus, we can reject Hypothesis 4.

### Pathway Model vs. Regression Model

In our previous analysis, we found that ITE had no direct effect on EC scores. Thus, it can be assumed that implicit theories of controllability of emotions were not involved in the system of relations of the studied variables. To test Hypothesis 5, we constructed two competing models with EC scales as dependent variables and Intelligence and Self-Esteem scores as predictors.

**Figure 4:**
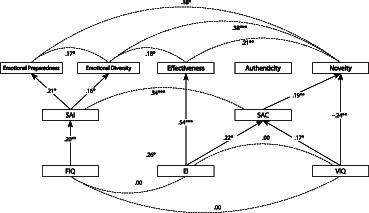
Intelligence, Emotional Intelligence, and Self-Assessed Intelligence and Creativity predict different domains of Emotional Creativity

We reduced both models to structural relations with significant coefficients obtained in the first step and evaluated the fit indices for both reduced models. Analysis results are presented in *Table 2.* The reduced pathway model demonstrated better fit indices for both c^2^-statistics and information criteria than the reduced regression model. Thus, we can assume that our Hypothesis 5, about more accurate reflection of the structure of relationships between the studied constructs by the model, with the inclusion of mediator effects of self-assessments, has been verified. The reduced pathway model with significant effects is presented in *Figure 4.*

**Table 2:** Pathway Model vs. Regression Model

Index	Regression full / Pathway full	Regression significant	Index delta for Regression significant model	Pathway significant	Index delta for Pathway significant model
c^2^	.924	42.28		36.458	
df(c^2^)	2	28		28	
*p*(c^2^)	.630	.041		.106	
AIC	4059.005	4048.361	-1.644	4044.539	-14.466
BIC	4247.829	4159.257	-88.572	4158.433	-89.396
SABIC	4048.458	4042.166	-6.292	4038.177	-1.281

## Discussion

This is the first comprehensive study of the relationship among Intelligence, Self-Assessment of Intelligence and Creativity, Emotional Creativity, and Implicit Theories of Emotions. Unlike other works, we were able to establish not only the direct contributions of both types of intelligence to EC, but also their contribution, through mediation of Self-Assessments of Intelligence and Creativity.

We found fewer associations of cognitive abilities with personality traits than expected. We do not support the allegedly closer relationship of crystallized (verbal) intelligence with personality, established using other questionnaires — in particular, the Big Five questionnaire (Rammstedt et al., 2020). Supporting Stankov’s (Stankov, 2018) move beyond the lexical approach in defining personality traits to establish connections between intelligence and personality and supporting the trend towards including EI in this connection (Colom et al., 2019), we implemented a transition to different levels of regulation — self-esteem and implicit theories.

Existing research suggests that EI should be associated with increased intelligence in problem solving — more active situation modification, reappraisal, and distraction control, but with less rumination (Bucich & MacCann, 2019; Peña-Sarrionandia et al., 2015). In our study, we did not find a significant increase in EI scores with an increase in intelligence. Additionally, we have not found any direct correlations between intelligence and EC scales except for the single negative correlation between VIQ and the Novelty scale. This finding corresponds to the assumption that people with high verbal intelligence may experience fewer new emotions (or distinguish them worse, which is manifested in questionnaire responses).

We accept Hypothesis 1 in terms of intelligence impact on self-assessed intelligence and creativity. Our data indicate that direct SAC, although associated with SAI, has its own domain specificity: SAI was predicted by fluid intelligence, while SAC was predicted by verbal and emotional intelligence.

We accept Hypothesis 2 — about the role of self-assessments as predictors of EC.

Our analysis of the indirect influence of intelligence on emotional creativity via SAI and SAC showed that self-esteem significantly mediates this relationship; thus, Hypothesis 3 is accepted. Moreover, SAI and SAC correspond to different domains of emotional creativity. Self-assessment of intelligence provided the association of Fluid Intelligence with Emotional Preparedness and Emotional Diversity, while self-assessment of creativity mediated the association between Verbal and Emotional Intelligence and Novelty in the emotion creation process. We also found that SAC provided a positive pathway from Verbal Intelligence to Novelty in Emotional Creativity, as opposed to a negative direct effect of Verbal Intelligence on the same Emotional Creativity component. This finding supports the assumption of the integrative role of a person’s self-awareness in the regulation of creative processes. It is the reflection of one’s intellectual abilities at the level of a person’s self-awareness that regulates Emotional Creativity to a greater extent than directly intellectual abilities and personality traits.

Our Hypothesis 4 about a mediation effect of Implicit Theories of Emotions in relationships between Emotional Intelligence and Emotional Creativity was rejected. We could not show any additional indirect effects of Emotional Intelligence on EC scales through implicit theories. Thus, the trait EI is an essential independent component of emotional Creativity manifestation in its effectiveness domain.

### An Integrative Model for the Regulation of Emotional Creativity by Different Types of Intelligence Allowed for a Clearer Understanding of the Relationships between Multi-Level Processes

The main theoretical assumption of the study was the inclusion of the level of self-awareness, represented by self-assessments of intelligence and creativity and implicit theories of emotions in the system of connections between different types of intelligence and emotional creativity.

In Emotional Creativity, we can distinguish domains with different levels of connection with different types of intelligence. The effectiveness of Emotional Creativity shows the closest direct relations with Emotional Intelligence. The authenticity of emotion creation seems to be the most independent from the Intelligence–Emotional Creativity domain. The novelty of the created emotions can be suppressed by higher levels of Verbal Intelligence, but at the same time can be maintained by higher Verbal Intelligence combined with Emotional Intelligence through self-assessment of creativity. Emotional preparedness, which is the capacity to understand and learn about one’s own and others’ emotions, and emotional diversity are linked to fluid intelligence through intelligence and self-esteem.

Thus, we can assume that the emotion creation process should be considered as a complex integrative phenomenon, with different domains regulated simultaneously by different types of intelligence through their reflection at the level of self-consciousness.

## Conclusion

We showed that the intelligence impact of emotional creativity was mediated by self-esteem in intelligence and creativity.

In relation to unity of intelligence and affect, we have given a strong argument for a multilevel integration of processes behind the interactions between cognitive and emotional spheres, as well as between more conscious and less conscious processes. We empirically supported the idea of the need for a multiple component analysis in each of the concepts under consideration — emotional intelligence, emotional creativity, implicit theories, and self-assessments of intelligence and creativity.

## Limitations

The main limitation of our study was the general participants’ interest in psychology due to their majoring in this field. Thus, further investigation on different samples is needed.

The obtained model indicators are not sufficiently strong, and improvements may be possible by expanding the samples. At the same time, these models help clarify inter-level transitions within the diversity of the studied processes, which conventional correlation analysis does not allow.

Data on the effects of direct self-assessments of intelligence and creativity on other studied variables allow us to consider them as a link between intellectual abilities, emotional creativity, and belief in the controllability of emotions. However, in our study, not all the participants took the ICAR intelligence test. And perhaps we would get more reliable results with the full design.

## Footnotes

1 The basis for such a data transformation was the understanding of regression residuals as a fraction of the variance of the corresponding variables not related to the variability of model predictors. Thus, when evaluating associations between variables for which gender and age effects have been established, the use of regression residuals allows us to analyze variances adjusted for general demographic effects.

## Appendix

**Table A:** Regression analysis by gender and age

Scale	F(df)	adj.p (F)^*^	adj. R^2^	Intercept			Gender=Males			Age			Age X Gender		
				ß	t	p(t)	ß	t	P(t)	ß	t	P(t)	ß	t	P(t)
*Intelligence*															
FIQ	0.59(3/148)	.966	< 0												
VIQ	2.38 (3/147)	.337	.03												
*Emotional Intelligence*															
Well-Being	1.01 (3/240)	.875	<.001												
Emotionality	0.68 (3/240)	.966	< 0												
Decreased Self-ontrol	0.40 (3/240)	.994	< 0												
Sociality	0.91 (3/240)	.875	< 0												
*Emotional Creativity*															
Authenticity	1.39(3/239)	.858	.005												
Effectiveness	0.16(3/239)	.994	< 0												
Emotional Preparedness	0.02 (3/239)	.997	< 0												
Novelty	2.82 (3/239)	.278	.02												
Emotional Diversity	7.80 (3/239)	<.001	.08	0.07	1.10	.274	-0.51	-2.97	.003	-0.25	-3.32	.001	0.16	1.16	.246
*Implicit Theories of Emotions*															
ITE	0.24(3/231)	.994	< 0												
*Self-Assessments*															
SAI	0.26 (3/236)	.994	< 0												
SAC	1.07(3/233)	.875	.001												

*Note: ^*^ Benjamini-Hochberg correction for Type I error*

**Table B:** Correlation analysis results

Scale		1	2	3	4	5	6	7	8	9	10	11	12	13	14
*Intelligence*															
1	FIQ	1.00													
2	VIQ	.26^**^ (.006)	1.00												
*Emotional Intelligence*															
3	Well-Being	.00 (.982)	-.15 (.154)	1.00											
4	Emotionality	-.03 (.827)	-.05 (.673)	-.46^**^ (5.33E-13)	1.00										
5	Decreased Self-Control	-.03 (.854)	-.02 (.886)	-.50^**^ (.000)	.43^**^ (4.80E-11)	1.00									
6	Sociality	-.11 (.314)	-.12 (.265)	.53^**^ (.000)	-.45^**^ (2.01E-12)	-.47^**^ (2.37E-13)	1.00								
*Emotional Creativity*															
7	Authenticity	.10 (.363)	-.01 (.953)	.08 (.348)	-.11 (.172)	.10 (.223)	.01 (.933)	1.00							
8	Effectiveness	-.10 (.348)	-.15 (.154)	.41^**^ (5.02E-05)	-.29^**^ (5.02E-05)	— 27^**^ (1.34E-04)	.39^**^ (3.64E-09)	.26^**^ (2.02E-04)	1.00						
9	Emotional Preparedness	.06 (.617)	.09 (.439)	.04 (.710)	-.05 (.598)	-.13 (.117)	.14 (.084)	-.02 (.858)	.24^**^ (8.41E-04)	1.00					
10	Novelty	-.05 (.630)	-.22^*^ (.019)	.07 (.442)	.02 (.819)	.04 (.720)	.17^*^ (.024)	-.01 (.956)	27^**^ (1.48E-04)	.28^**^ (8.95E-05)	1.00				
11	Emotional Diversity	-.08 (.495)	.00 (.995)	-.04 (.665)	.05 (.568)	.12 (.125)	.03 (.758)	.09 (.302)	.21^**^ (.005)	.25^**^ (4.82E-04)	.54^**^ (.000)	1.00			
*Implicit Theories of Emotions*															
12	ITE	-.01 (.933)	-.15 (.154)	.12 (141)	-.07 (.434)	— 27^**^ (1.46E-04)	.21^**^ (.006)	-.03 (.749)	.19* (.010)	.04 (.665)	.08 (.348)	-.03 (.765)	1.00		
*Self-Assessments*															
13	SAI	.25^**^ (.007)	.10 (.363)	.16^*^ (.046)	-.04 (.665)	-.16^*^ (.046)	.23^**^ (.001)	.04 (.710)	.11 (.182)	.18^*^ (.019)	.13 (.115)	.16^*^ (.033)	-.01 (.953)	1.00	
14	SAC	.13 (.224)	.18 (.083)	.11 (.175)	-.07 (.434)	-.12 (.137)	.22^**^ (.002)	.02 (.878)	.10 (.262)	.09 (.314)	.19^**^ (.010)	.14 (.083)	-.02 (.886)	.43^**^ (8.55E-11)	1.00

*Note: the corrected for Type I error p-value of the Pearson correlation coefficient is presented in parentheses; ^*^ indicates p-value < 0.05, ^**^ indicates p-value < 0.01*

**Table C:** Intelligence and Emotional Intelligence predict EC, SAI, and SAC

Scale	F(df)	adj.p (F)^*^	adj. R^2^	Intercept			FIQ			VIQ			El		
				ß	t	P(t)	ß	t	P(t)	ß	t	P(t)	ß	t	P(t)
*Emotional Creativity*															
Authenticity	0.90 (3/147)	.515	<0												
Effectiveness	13.37 (3/147)	6.44E-07	.20	-0.03	-0.41	.685	-0.07	-0.84	.403	-0.10	-1.27	.207	.51	5.96	1.93E-08
Emotional Preparedness	0.63 (3/147)	.594	< 0												
Novelty	2.80 (3/147)	.074	.03												
Emotional Diversity	1.30 (3/147)	.389	.01												
*Self-Assessments*															
SAI	4.44 (3/146)	.018	.06	0.02	0.28	.777	0.22	2.80	.006	0.05	.061	.540	0.16	1.97	.051
SAC	4.03 (3/144)	.020	.06	-0.01	-0.16	.872	0.08	0.94	.351	0.17	2.08	.040	0.22	2.50	.014

*Note: ^*^ Benjamini-Hochberg correction for Type I error*

**Table D:** SAI and SAC predict EC

Scale	F(df)	adj.p (F)^*^	adj. R^2^	Intercept			SAI			SAC			SAI X SAC		
				ß	t	P(t)	ß	t	P(t)	ß	t	P(t)	ß	t	P(t)
*Emotional Creativity*															
Authenticity	1.61 (3/231)	.189	.01												
Effectiveness	2.48 (3/231)	.078	.02												
Emotional Preparedness	2.61 (3/231)	.078	.02												
Novelty	4.87 (3/231)	.013	.05	-0.05	-0.71	.478	0.08	1.17	.243	0.177	2.50	.013	0.140	2.21	.028
Emotional Diversity	3.20 (3/231)	.060	.03												

*Note: ^*^ Benjamini-Hochberg correction for Type I error*
